# Effects of Mindfulness Meditation on Doctors’ Mindfulness, Patient Safety Culture, Patient Safety Competency and Adverse Event

**DOI:** 10.3390/ijerph19063282

**Published:** 2022-03-10

**Authors:** Chao Liu, Hao Chen, Xinyi Cao, Yini Sun, Chia-Yih Liu, Kan Wu, Yu-Chao Liang, Szu-Erh Hsu, Ding-Hau Huang, Wen-Ko Chiou

**Affiliations:** 1 School of Journalism and Communication, Hua Qiao University, Xiamen 361021, China; victory666666@126.com (C.L.); syn-1989@163.com (Y.S.); 2 Business Analytics Research Center, Chang Gung University, Taoyuan 33302, Taiwan; haochen19606@163.com (H.C.); kan@gap.cgu.edu.tw (K.W.); 3 School of Film and Communication, Xiamen University of Technology, Xiamen 361021, China; 4 Clinical Neurocognitive Research Center, Shanghai Key Laboratory of Psychotic Disorders, Shanghai Mental Health Center, Shanghai Jiao Tong University School of Medicine, Shanghai 200030, China; rekixinyicao@163.com; 5 Department of Psychiatry, Chang Gung Memorial Hospital, Taipei 10507, Taiwan; liucy752@cgmh.org.tw; 6 Department of Industrial Design, Chang Gung University, Taoyuan 33302, Taiwan; ycliang465264@gmail.com (Y.-C.L.); h410@hotmail.com (S.-E.H.); 7 Institute of Creative Design and Management, National Taipei University of Business, Taoyuan 22058, Taiwan; hau1012@gmail.com

**Keywords:** mindfulness meditation, mindfulness, patient safety culture, patient safety competency, adverse event

## Abstract

Objective: This study investigated the effects of mindfulness meditation on doctors’ mindfulness, patient safety culture, patient safety competency, and adverse events. Methods: We recruited 91 doctors from a hospital in China and randomized them to mindfulness meditation group (*n* = 46) and a waiting control group (*n* = 45). The mindfulness meditation group underwent an 8-week mindfulness meditation intervention, while the control group underwent no intervention. We measured four main variables (mindfulness, patient safety culture, patient safety competency, and adverse event) before and after the mindfulness meditation intervention. Results: In the experimental group, mindfulness, patient safety culture and patient safety competency were significantly higher compared with those of the control group. In the control group, there were no significant differences in any of the three variables between the pre-test and post-test. Adverse events in the experimental group were significantly lower than in the control group. Conclusions: The intervention of mindfulness meditation significantly improved the level of mindfulness, patient safety culture and patient safety competency. During the mindfulness meditation intervention, the rate of adverse events in the meditation group was also significantly lower than in the control group. As a simple and effective intervention, mindfulness meditation plays a positive role in improving patient safety and has certain promotional value.

## 1. Introduction

Patient safety is the core indicator to measure the quality of medical care. However, in recent years, the global patient safety situation has become worrisome, and adverse events caused by medical care errors occur frequently [1]. According to WHO statistics, the incidence of adverse events in hospital patients around the world is 10% on average. In developing countries, the possibility of adverse events is much higher than that in developed countries due to the limitations of medical level and resources [2]. Adverse events can lead to longer hospital stays, higher medical costs, physical disability and even death. As a result, improving patient safety is now a global movement [3]. Studies show that 50% of adverse events can be prevented. The existing human factors project studies on patient safety have focused on physiological human factors, cognitive human factors and organizational perspectives. Previous studies on patient safety have identified factors that influence patient safety, including burnout, nursing education levels, attrition of nursing staff and inability to perform nursing tasks [4]. However, few studies have investigated the role of patient safety competency of healthcare personnel in patient safety, indicating the first gap in previous studies. Under the promotion of the WHO, various countries have successively integrated the Patient Safety Tutorial Guide compiled by the WHO into clinical medicine, nursing and other professional teaching plans [5,6]. However, patient safety education ignores the impact of mindfulness on safety competency of medical workers, indicating a second gap in previous studies. To fill these gaps, this study explored the impact of mindfulness on health care providers’ level of mindfulness, safety competency and patient safety. Filling these gaps could provide new ways to improve patient safety.

### 1.1. Patient Safety

Patient safety is critical to the delivery of health services, and adverse events, such as adverse drug events, and can cost a 700-bed hospital $5.6 million. Better patient safety helps reduce hospital stays, costs and mortality [7]. Such evidence demonstrates the importance of patient safety in health services. Near-misses that are not fully reported and recorded in hospital records in certain patient safety measures offer good lessons for improving patient safety. Therefore, adverse events and near-misses should be included when assessing patient safety [8]. Laschinger demonstrated the usefulness of using self-reported data to measure patient safety. Healthcare workers were asked to report the frequency of patient falls, hospital-acquired infections, medication errors and patient complaints over the past year [9]. When anonymity is ensured, subjective data can encourage health care providers to fully report the frequency of incidents that threaten patient safety, and other recent studies have used this subjective measure [10]. Patient safety is often related to staffing, especially in highly-dependent intensive care settings. One possible explanation for this finding is that staffing is directly related to the number of hours of care each patient receives each day, which is also related to patient safety. Direct healthcare time can increase nursing communication with patients, which also contributes to patient safety [10,11]. Recent studies have further shown that patient safety outcomes are positively correlated with patient-centred care, safety risk management, and evidence-based medical practice [12]. Further evidence suggests that patient safety culture, clinical practice, and continuous quality improvement improve patient safety [13]. This evidence suggests that patient safety competency of health care providers may contribute to patient safety.

Patient safety culture is the common concept, attitude, value and behaviour of medical staff formed by medical institutions in the process of ensuring patient safety. It can also be understood as Hippocrates’ motto “first do no harm”, which integrates “safety” into every unit of the organization and infuses it into every operating norm, thus elevating safety to the top priority [14]. The China Hospital Association has officially listed the construction of patient safety culture as one of the ten goals of patient safety. The establishment of patient safety culture can effectively avoid or reduce the occurrence of errors and accidents [15]. At the same time, medical staff’s awareness of patient safety culture is positively correlated with the reduction of adverse medical events. Increasing medical staff’s awareness of patient safety culture can effectively guarantee patient safety [15]. Patient safety culture can positively guide the thought and behaviour of medical staff, and is the internal power to reduce adverse events and realize patient safety [16]. Studies have shown that the evaluation of patient safety culture is the premise of finding patient safety problems, improving an employee’s patient safety awareness, formulating improvement measures, and promoting the construction of patient safety culture [17]. Han believed that establishing patient safety culture was an important means to evaluate the quality of medical care, identify and prevent errors and accidents, and was the basis for ensuring the safety of medical care [18]. Human factors should not only strengthen the construction of safety culture from the organization, but also strengthen the safety competence of medical staff from the personal point of view [1]. Patient safety competency is closely related to the occurrence of adverse events in hospitals. Strengthening the training of patient safety competency for medical staff can effectively reduce the occurrence of adverse events and improve the quality of medical services [19].

### 1.2. Patient Safety Competency

Patient safety competency refers to the cognition, skill and attitude that medical staff possess in the risk control process of avoiding or reducing unsafe factors in the health care environment and preventing patients from suffering accidental injuries in order to ensure patient safety [18]. The institute of medicine (IOM) points out that the college education received by clinical medical workers does not include enough or provide continuing education to deal with patients with diversified needs. As a result, they put forward five points that characterize the competence of health-related workers: patient-centred care, communication and cooperation in an interdisciplinary team based on evidence-based medical practice, the promotion of quality and the effective use of information [20]. The “quality promotion” aspect includes the “identification of failures and risks in the health care environment; the understanding and implementation of basic safety design principles (e.g., safety standardization principles) [5]; the continuous understanding and measurement of the quality of care related to patients and society from the perspective of structure, process and results; to improve the health care system and improve the process of health care by designing intervention measures and testing the effects of intervention, so as to achieve the goal of improving the quality of medical care”. These descriptions are closely related to patient safety [21]. This emphasis is also reflected in the firm and close combination of patient safety competency and professional education. Patient safety education for health related professionals has become the focus of health care education around the world, and medical and nursing majors have begun to carry out patient safety education in higher education [22]. Australia, the United Kingdom, the United States and other developed countries have begun to discuss how to improve medical undergraduate professional education to promote patient safety earlier. At present, a total of 11 medical colleges and universities around the world have participated in the “Medical Undergraduate Patient safety education” project promoted by the WHO World Alliance for Patient Safety [19]. The Chinese Ministry of Health commissioned the Chinese Hospital Association to translate into Chinese the multidisciplinary edition of Patient Safety Tutorial Guide compiled by the World Health Organization [23]. In succession, clinical medicine, nursing and other majors integrate the patient safety curriculum guide into their professional teaching plan, the aim of which is to establish “safety first” professional values, cultivate the students’ professional risk consciousness, communication ability and team cooperation ability, guides the student to respect life and respect patients, attaches great importance to the rules, regulations, and technical specifications and to treat mistakes and injuries appropriately and to learn from them [24].

However, patient safety education is not only about mastering medical professional knowledge, so the classroom teaching method in traditional education far from meets the requirements of improving patient safety competency, and the performance of individual clinicians is still a key and largely unsolved factor of patient safety competency [25]. Individual performance is important for diagnosing errors, but they are hard to measure and are rarely systematically studied. Diagnostic errors often result from cognitive biases, also known as “cognitive responsiveness tendencies” (CDRs). Because CDRs are such an integral part of human cognition, they are difficult to overcome [26]. Chan has outlined more than 30 CDRS and proposed a “de-skewing strategy” to prevent errors caused by CDRS. The most prominent of these strategies is metacognition or an individual’s knowledge of his or her own thought processes [27]. It is worth noting that there is a significant overlap between metacognition and mindfulness, non-judgmental awareness of the present. Increased mindfulness leads to greater awareness and understanding of one’s experiences, including thoughts, emotions, and bodily sensations. Because education can improve mindfulness, mindfulness coaching for clinicians holds promise as a strategy for reducing diagnostic and other medical errors [28].

### 1.3. Mindfulness

Kabat-zin defines mindfulness as a kind of awareness that springs from a purposeful focus on the present moment and an unjudgmental awareness of the experience as it unfolds from moment to moment. At its core, mindfulness is about two things: focusing your attention on the present moment [29] and not to evaluate all the concepts presented at the moment. Developing an awareness of the here and now and maintaining an open and receptive attitude is important. Non-judgment means not blaming yourself, your environment, or others, which is necessary for being fully aware of your current psychosomatic feelings or experiences [30].

Over the past 20 years, researchers have used mindfulness for therapeutic purposes in clinical populations, finding that it can reduce stress, anxiety, pain and depression. In addition, researchers applied mindfulness to non-clinical populations and found that mindfulness improves mental health, quality of life, and subjective well-being [31]. Recent studies have shown the impact of mindfulness-based intervention guidance on occupational mental health of medical staff. Health care providers are using mindfulness practices to address mental and physical stress, emotional distress and burnout, while improving empathy, job satisfaction and well-being [32,33,34].

Humans are often more sensitive to negative things than to positive ones; for example, people are more sensitive to danger, referred to in psychology as negativity bias, which makes people feel stressed and leads to anxiety and even depression [35]. The principle behind mindfulness stress reduction is to help people get rid of negativity bias by awareness and non-judgment. Therefore, when we enter a state of mindfulness, where we accept all our thoughts and feelings without judgment, and can harness our attention to the present moment, our stress and anxiety caused by negative bias will be reduced [36].

Previous research has shown that mindfulness has positive effects on executive function, attention and awareness. However, little is known about the use of mindfulness in healthcare education [37]. Specifically, there are knowledge gaps about the impact of mindfulness on healthcare practice, particularly around the possible impact on patient safety. Some scholars have proposed that increasing the mindfulness of clinicians can improve the safety competence of medical staff and reduce medical errors, but there is no empirical test for this hypothesis [28].

Based on the above arguments and evidence, we propose the following hypotheses:

**Hypothesis** **1** **(H1).** *Mindfulness meditation can significantly improve subjects’ level of mindfulness, patient safety culture and patient safety competency*.

**Hypothesis** **2** **(H2).** *The rate of adverse events in the mindfulness meditation group was significantly lower than that in the control group*.

## 2. Methods

### 2.1. Participants

The participants of this study were doctors working in a hospital in China. We advertised for recruitment through the hospital intranet. A final total of 91 eligible participants were recruited; the demographic information of the participants is presented in Table 1. The participants were randomly divided into two groups: the mindfulness meditation (MM) group (*n* = 46) and the waiting control group (*n* = 45). The differences in demographic factors, such as age composition and sex ratio composition between the two groups, were not statistically significant.

### 2.2. Instruments

The Health Professional Education in Patient Safety Survey (H-PEPSS). This scale was developed by Ginsburg et al. in 2012 to evaluate the safety competency of doctors according to the six items of a social and cultural basic framework of patient safety, proposed by WHO and relevant international occupational institutions [38]. The scale includes 23 items in six dimensions, including work team, identification and response to safety adverse events, effective communication, safety risk management, safety culture and understanding human and environmental factors. The scale is self-rated, and the Likert 5 scoring method is applied. The higher the score, the higher the perception degree and demand of the patient safety knowledge ability of the respondent. The Chinese version of the PEPSS is used to measure doctors’ safety competency [39]. Previous studies have shown that the PEPSS has good validity. In this study, Cronbach’s alpha was 0.91. 

The Mindfulness Attention Awareness Scale (MAAS). This is a 15-item assessment instrument that tends to extract the status of the subjects, with questions and answers ranging from 1 (not at all) to 5 (very good) [40]. The score shown in the scale demonstrates the degree of the subject’s mindfulness level. This instrument was very effective at evaluating subjects’ subconscious and non-judgmental perceptions, surpassing similar scales used to evaluate attentive concentration and current consciousness. The Chinese version of MAAS is used to measure doctors’ mindfulness [41]. Previous studies have shown that the MAAS has good validity. In this study, Cronbach’s alpha was 0.89.

Hospital Survey on Patient Safety Culture (HSOPSC). This scale was developed by the American Agency for Healthcare Research and Quality and measures teamwork within the department, managers’ expectations and actions to promote safety, organizational learning and continuous improvement of patient safety, management support, the overall feeling of patient safety, the error of feedback and communication, communication openness, event reporting frequency, coordination between departments, staffing, shift security and response to the error of punitive 12 dimensions, which consist of a total of 42 items and has good reliability and validity. The Likert 5 scoring method was adopted for each item, and the degree of agreement was selected from strongly disagree (1 point) to strongly agree (5 points). The higher the score was, the better the cognition level of doctors on patient safety culture [42]. The Chinese version of the HSOPSC was used to measure doctors’ patient safety [43,44]. Previous studies have shown that the HSOPSC has good validity. In this study, Cronbach’s alpha was 0.92.

Adverse events. We examined six types of adverse medical events that frequently occur in hospitals: medication errors, patient falls, patient injuries, nosocomial infections, delayed care by others, and incomplete documentation [45]. Using a Likert 7 points scale ranging from 0 (daily) to 7 (never), all recent physician-perceived adverse events were rated for frequency. Responses were collected to indicate the frequency of adverse events as follows: daily, several times a week, once a week, several times a month, once a month or less, and never. In addition, a higher score indicated a lower frequency of adverse events [46].

### 2.3. Mindfulness Meditation Intervention

The MM intervention in this study was delivered through a 90 min group training session. Each session consisted of three consecutive phases: (1) 15 min of psychoeducation covering topics such as introduction to MM; (2) 30 min of MM exercises; and (3) a final discussion phase lasting 45 min, where participants could share their MM experiences with other participants and the instructor. MM interventions need to be practiced in a quiet environment. The general procedure is to look inwards, focus on breathing and remove all external distractions [47] by directing attention to the flow of breathing, being aware of breathing but not trying to control it, accepting and observing sensations and feelings without avoiding or trying to control them [48]. When experiencing an uncomfortable feeling and thoughts such as “I cannot stand it”, subjects were asked to not immediately respond to them as facts, but try to notice them and from time to time observe them as one can observe one’s own internal experience without being influenced by it [49]. Finally, participants were asked to practice mindfulness between sessions and to use the techniques of mindfulness in their workplace [50,51].

### 2.4. Procedure and Design

**Figure 1:**
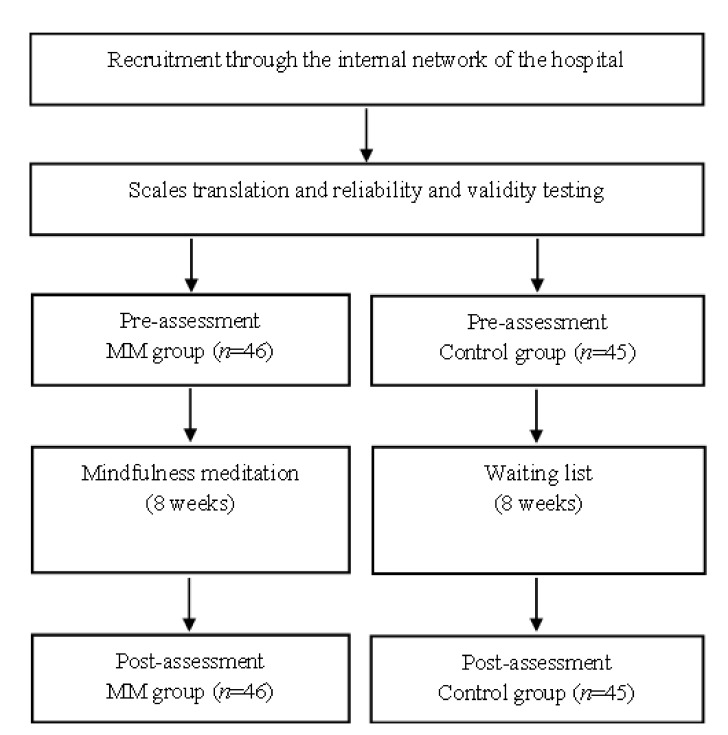
Procedure flow chart.

We advertised our MM recruitment on the intranet of a hospital in China, describing MM as a self-exploration activity designed to help doctors better understand themselves and solve current problems. Doctors who were interested and eligible to participate in our MM study provided their registration information. Inclusion criteria were: (1) having been a doctor for at least 3 years and (2) speaking Chinese and being able to complete the questionnaire. Exclusion criteria were: (1) self-reported diagnosis of depression, anxiety disorder, bipolar disorder, substance abuse or attempted suicide by a healthcare professional, and (2) previous experience in MM. We then randomly assigned 91 eligible participants to either the experimental group (i.e., MM intervention group, 46 participants) or the control group (i.e., waiting group, 45 participants). The study was conducted by researchers with 10 years of practical MM experience and 2 years of MM teaching experience. The meeting was held in a quiet, unpretentious room and each participant was given 50 yuan (Chinese dollars) at the beginning of the survey to motivate them. Instructions were provided to participants at the start of the survey to ensure they understood them before proceeding. After confirming that they understood the instructions, participants provided demographic information and completed the following questionnaires (pre-tests): (1) the Mindfulness Attention Awareness Scale (MAAS); (2) the Patient Safety Culture Scale (HSOPSC); (3) the Patient Safety Competence Scale (PEPSS); and (4) the Adverse Events Scale (MAE). The time required to answer the questionnaire was approximately 20 min. After all participants completed the pre-assessment, they were randomly assigned to either the MM group or a control group based on a computer-generated randomiser matched to the gender variable. The MM group received the MM intervention 3 times a week for 8 weeks, while the control group did not receive any intervention. Many previous studies on MM have used an intervention period of 8 weeks [47,48,49]. So, this study referred to these studies and set the total length of the intervention to 8 weeks. At the end of the intervention period (week 8), participants completed the same questionnaire again (post-assessment) and received an additional 50 yuan as compensation (as shown in Figure 1).

This study followed a funnel debriefing procedure. Participants were asked if they knew the purpose of the study and the topic being investigated, and if they were aware of the same questions in the pre-test and post-test. In order to recruit subjects who were oblivious to the experimental conditions and naively performed the MM exercise, the funnel debriefing helped to obtain a homogeneous sample in both groups. Participants were given the opportunity to record any number of questionnaires anonymously and could withdraw at any stage. The study was approved by the Ethics Committee of Chang Gung University (IRB No. 202001014B0D001) and the protocol was carefully reviewed to ensure that it complied with the ethical norms of the Chinese Psychological Association.

### 2.5. Data Analysis

SPSS 22 software was used for analysis of variance (ANOVA). The significance level was set to 0.05.

## 3. Results

**Figure 2:**
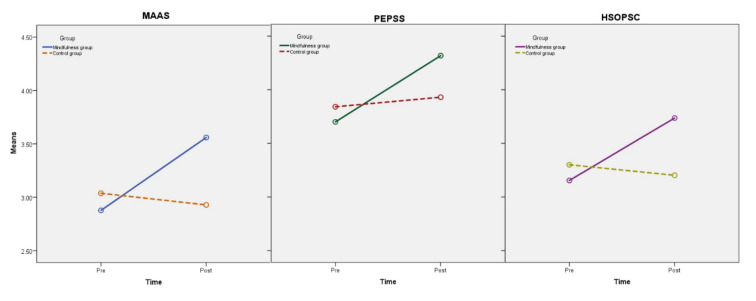
Interaction between time and group for each measure.

This study conducted three 2 (Time: pre, post) × 2 (Group: MM, control) ANOVA with repeated measures on mindfulness (MAAS), patient safety culture (HSOPSC) and patient safety competency (PEPSS). The *p*-values of Box’s test and Mauchly’s test were all greater than 0.05 and showed that the observed covariance matrices of the dependent variables are equal across groups, indicating that these data were suitable for ANOVA. The descriptive statistics are presented in Table 2, and the results of the ANOVA are presented in Table 3 and Figure 2.

According to the results of ANOVA, mindfulness meditation can significantly improve doctors’ mindfulness, patient safety culture and patient safety competency. For patient safety culture and patient safety competency, although the main effect of the group was not significant, the main effect of the time was significant, and the time ×group interaction effect was significant (see Table 2 and Figure 2), indicating that mindfulness meditation has a significant promoting effect on them. Hypothesis 1 is supported.

We also collected data on medical adverse events (MAE, see Table 1) in both groups during the intervention period (8 weeks). Independent *t*-test results showed significant differences (*p* = 0.003, Std. error = 0.087) in MAE scores between the two groups. The MAE score in the MM group was significantly higher than that in the control group, indicating a lower rate of adverse events. Hypothesis 2 is supported.

## 4. Discussion

Mindfulness is a practice that originated in Buddhism and encourages non-judgmental awareness of current thoughts, emotions, and physical feelings to achieve the ability to free oneself from worry [52]. With the development of research on mindfulness therapy in the field of heart psychology, mindfulness is becoming more and more popular, and also more and more de-religious [53]. Regarding the Buddhist elements of mindfulness, Kabat-Zinn argues that mindfulness is not Buddhism, but the essence of Buddhism. Mindfulness is therefore considered universal and compatible with science [54]. These Buddhist connections allow practitioners of mindfulness to enjoy the Buddha’s legacy while avoiding any unwanted religious connotations [55]. Similarly, when mindfulness is declared universal, it seems to be less about Buddhism and more about a “basic human capacity”. Mindfulness has become more secular and less religious, with scientific testing promoting its clinical and educational applications [56]. Advocates of mindfulness have also deliberately downplayed the Buddhist aspect of mindfulness, promoting it by translating Buddhist ideas into scientific and secular language [57]. With the continuous development of mindfulness, its application is becoming more and more widespread [58]. This study introduces mindfulness as a human factor of psychological cognition into the intervention activities of medical safety, and further broadens the application scope of mindfulness.

### 4.1. Mindfulness and Safety Culture

There were significant differences in patient safety culture scores before and after the test. The reason for the improvement in patient safety culture is that employees internalize the safety requirements of the organization as their own safety goals after the mindfulness exercise, and realize the improvement in patient safety culture by actively inhibiting their own unsafe behaviours [59]. Mindfulness can further strengthen self-control and mental functioning by fostering open attention and awareness to present realities and experiences. One mindfulness-based intervention has been shown to increase self-control [60]. In the aspects of positive and objective facing, attention control, acceptance and non-judgment, mindfulness can effectively relieve the depletion of self-control and improve the level of self-control. Mindfulness awareness plays a very important role in the cognitive process of self-control, and it plays an important cognitive role in the subject’s state response and behaviour response in the process of self-control [61]. Mindfulness training focusing on the present to highlight the focus of present control, including emotional control, pays attention to the control of training; the training will help ease the ego depletion, so the effective control of attention in mindfulness by avoiding the ego depletion in self-control will improve self-control ability; that is to say, a person’s level of mindfulness can self-control level forecast [62]. In the attrition state, individuals are less able to control their cognition, emotions, decisions, and behaviours, which are critical to maintaining a high level of safe behaviour. People with high levels of self-control tend to be disciplined, reliable and exhibit more safe behaviours at work [63]. Past research has also shown that improving self-control at work can effectively reduce intentional violations and other unsafe behaviours. At work, the negative consequences of unsafe behaviour sometimes do not show up in time [64]. Unsafe behaviour is often not in accordance with the rules to choose a shortcut, or in order to save trouble, for comfort not in accordance with the requirements of the operation. The benefits of these unsafe behaviours are easily perceived by individuals [64]. Therefore, hospitals usually adopt a series of control procedures to control the unsafe behaviour of employees, standardize the behaviour of employees, and improve the hospital patient safety culture [65].

### 4.2. Mindfulness and Safety Competencies

There were significant differences in safety competency scores of doctors before and after the test. On the one hand, mindfulness can improve doctors’ safe behaviour by reducing the tendency to take shortcuts that may lead to disciplinary violations. On the other hand, mindfulness can improve doctors’ ability to consciously think. Employees with high levels of mindfulness tend to be reluctant to simplify their view of everything they encounter for the first time, and do not blindly follow the opinions of others [66]. Recent studies have found that mindfulness improves job satisfaction, intrinsic motivation, and reduced emotional burdens, which are also important predictors of safety competency [62]. Mindfulness may be especially important for those in the healthcare industry. In complex medical work, doctors ignore seemingly small problems and unconsciously imitate unsafe behaviours of others, which can lead to adverse events [67]. Mindfulness, on the other hand, often helps doctors become more alert and aware of changes around them, forming the habit of independent observation and judgment [68]. The safe behaviour of doctors is controlled by two basic cognitive systems: (1) the unconscious, autonomous and effortless processing system, which is driven by intuition and past experience and runs at a high speed; (2) a conscious, controlled, laborious processing system that uses thought to reason and operates in a slow and continuous manner [69]. That is to say, problems in the use of dual process systems may undermine the safety behaviour of employees. In previous studies, researchers have found that mindfulness improves safety behaviour by improving the use of dual systems [70]. As Brown’s analysis points out, mindfulness is associated with metacognition and executive attention, which can modulate dual systems in positive ways. Mindfulness enables employees to better focus on the present moment and process information from external stimuli and internal mental states in a deeper and more open way, thus avoiding or reducing the occurrence of unsafe behaviours [71]. On the one hand, mindfulness can promote employees to become more aware of internal experiences and intuitions that they might not otherwise pay attention to, and people with such awareness are more likely to learn from them continuously, thus improving their safety competence [72]. There is evidence that mindfulness training, and higher levels of mindfulness, can improve situational awareness and provide a good foundation for safe behaviour in high-risk industries such as healthcare. “Pay attention” can improve employee safety behaviour by diluting the effect that safety behaviour may be influenced by an inherent desire to take shortcuts [73]. In addition, recent studies have shown that mindfulness improves job satisfaction, intrinsic motivation, and reduced emotional burdens, which are also important predictors of safety competency. Mindfulness, on the other hand, can improve one’s ability to think consciously [28]. The researchers believe that conscious thinkers are more likely to be reluctant to simplify their view of everything they encounter for the first time. This is particularly important for security in high-risk industries, where ignoring seemingly small problems can be disastrous. In addition, studies have shown that people who are consciously thinking are less likely to be affected by negative emotional burdens [64].

### 4.3. Mindfulness and Adverse Events

The adverse events in the experimental group were significantly lower than those in the control group. Practicing mindfulness does improve doctors’ ability to regulate and focus. Especially during the initial stages of practicing mindfulness, participants actively focus on the present experience and can cope with distracting and mind-wandering thoughts [74]. In reality, the mind is always interacting with the external environment. On the one hand, mindfulness can further strengthen individuals’ self-control and psychological function by cultivating their open attention and awareness of current reality and experience. On the other hand, mindfulness can help individuals improve their self-control level in the aspects of positive and objective facing, attention control, acceptance and non-judgment. Existing studies have shown that mindfulness-based training can effectively improve attention [75]. Doctors work in a more complex environment than the average, and as a result, doctors sometimes simply cannot focus on every situation and recognize important changes in time that can significantly reduce the incidence of adverse events [76]. Functional magnetic resonance imaging (FMRI) studies have shown that mindfulness is associated with activation of the amygdala, suggesting that people who correct their thoughts have a greater impact on concentration and attention control, which in turn reduces adverse events [77]. Therefore, it is necessary to organize employees to improve their awareness through mindfulness learning so as to avoid negative effects of emotions, which is necessary to reduce adverse events. Based on the dual process model of human cognition, Zhang et al. investigated the effects of mindfulness on safety behaviour and boundary conditions of operators [78]. In a survey of nuclear plant control room operators, mindfulness was found to be inversely associated with adverse events. Controlling for age, intelligence, work experience and conscientiousness, the effect remained significant. In addition, the results showed that mindfulness had a greater impact on adverse events among experienced or more intelligent operators [79]. This study theoretically understands the benefits of mindfulness in reducing adverse events. In practice, it provides a new, effective standard that can be used in physician selection and training programs to improve patient safety. In daily life, mindfulness also has a positive impact on personal safety behaviours [80]. Greg Feldman et al. studied whether the difference in young adult drivers’ mindfulness is related to unsafe behaviours, such as texting while driving, and the results showed that people with lower mindfulness level send text messages more frequently while driving. The results could help develop mindfulness-based interventions to prevent unsafe texting while driving [81]. Taken together, it seems that people with higher levels of mindfulness are more likely to have lower adverse events.

### 4.4. Research Limitations and Future Studies

This study combined mindfulness and patient safety and explored the mechanism of mindfulness’s influence on patient safety, which has some innovative significance, but there are still deficiencies. (1) Self-rating scale is used for evaluation. Due to the existence of social approval effect, doctors may underreport the frequency of occurrence of accident signs when conducting self-evaluation, which reduces the variation of safety results and thus weakens the relationship between prediction variables and outcome variables. (2) In this study, the method of testing is group testing. During the testing, the researcher pays great attention to the rigor of the testing procedure, but there are still some subjects who do not fill in the questionnaire according to the instructions (for example, they answer the questionnaire first without listening to the instructions). In addition, the speed of each person to answer the questionnaire is different. It is suggested that group testing can be avoided in future studies to reduce interference between subjects. (3) This study takes hospitals as the research field, and the subjects are limited to doctors, so the research results cannot be extrapolated to other fields. Therefore, whether the research results can be extrapolated to non-hospital situations is worth further discussion.

This study discusses the influence of mindfulness on patient safety. However, patient safety is affected by a variety of complex factors, and further research and discussion are needed. In view of the shortcomings of this study, the following research prospects are proposed: (1) According to the nature of variables, future studies can investigate the influence of mindfulness level on doctors’ situational awareness and safe operation behaviour from the perspective of state. (2) For the study design, future studies should include a horizontal design to explore the factors influencing doctors’ mindfulness. (3) For data measurement methods, future studies should integrate multiple data sources, such as evaluation by others and behavioural observation, to test the validity of the results of this study. (4) Research on mindfulness can be further extended to all walks of life in the future, so that mindfulness can play an important role in improving the safety behaviour of employees. (5) Increase the number and coverage of survey samples, improve research methods, and develop localized scales applicable to the domestic environment to make the survey results more convincing and universal. (6) Different types of mediating variables can be considered in subsequent studies to further reveal the mechanism of mindfulness on patient safety.

## 5. Conclusions

The intervention of mindfulness meditation significantly improved the level of mindfulness, patient safety culture and patient safety competency. During the mindfulness meditation intervention, the rate of adverse events in the meditation group was also significantly lower than in the control group. As a simple and effective intervention, mindfulness meditation plays a positive role in improving patient safety and has certain promotion value.

## Figures and Tables

**Table 1:** Demographic characteristics of participants.

Characteristic	Total	MM Group	Control Group
Age (SD)	39.71 (7.82)	38.42 (8.13)	41.03 (6.54)
Male (%)	37 (41%)	19 (41.3%)	18 (40%)
Female (%)	54 (59%)	27 (58.7%)	27 (60%)

Note. No demographic characteristic was significantly different among the two groups.

**Table 2:** Descriptive statistics.

Group	Measure	Mean (SD)	
		Pre	Post
MM	MAAS	2.878(0.765)	3.560(0.748)
	PEPSS	3.701(0.880)	4.318(0.908)
	HSOPSC	3.154(0.838)	3.737(0.850)
	MAE		5.065(0.437)
Control	MAAS	3.038(0.837)	2.929(0.727)
	PEPSS	3.842(0.953)	3.931(0.771)
	HSOPSC	3.301(0.843)	3.202(0.803)
	MAE		4.804(0.391)

**Table 3:** ANOVA Results.

Measure	Variable	F	*p*	η^2^
MAAS	Time **	6.753	0.011	0.071
	Group *	3.976	0.049	0.043
	Time × Group ***	12.875	<0.001	0.126
PEPSS	Time **	8.813	0.004	0.090
	Group	0.761	0.385	0.008
	Time × Group *	4.928	0.029	0.052
HSOPSC	Time *	4.063	0.047	0.044
	Group	2.332	0.130	0.026
	Time × Group **	8.017	0.006	0.083

Note. * *p* < 0.05; ** *p* < 0.01; *** *p* < 0.001.

## Data Availability

The datasets during the current study are not publicly available due to privacy restrictions but are available from the first author on reasonable request.
